# A Study of the Profile of Scrub Typhus in a Tertiary Care Hospital in Jharkhand: An Underestimated Problem

**DOI:** 10.7759/cureus.26503

**Published:** 2022-07-01

**Authors:** Sangita D Kamath, Sarita Kumari, Ashok Sunder

**Affiliations:** 1 Internal Medicine, Tata Main Hospital, Jamshedpur, IND

**Keywords:** orientia tsutsugamushi, doxycycline, rash, eschar, typhus, fever

## Abstract

Background and objective

Scrub typhus (ST) is a rickettsial infection caused by *Orientia tsutsugamushi*, which is transmitted by the bite of the larval stage (chiggers) of trombiculid mites. Although it presents as an acute undifferentiated febrile illness (AUFI), its course can be complicated with acute respiratory distress syndrome, acute kidney injury (AKI), myocarditis, meningoencephalitis, hepatitis, multi-organ dysfunction syndrome (MODS), and ultimately death. This study aimed to evaluate the epidemiological features, clinical profile, laboratory features, and clinical outcomes of cases of scrub typhus and identify the predictors of disease severity.

Methods and materials

This study is a retrospective observational study that included confirmed cases of scrub typhus admitted in the medical wards and critical care unit (CCU) of Tata Main Hospital (TMH) from January 1, 2019, to December 31, 2021. The case records of patients were analyzed for demographic characteristics, clinical features, treatment, and outcomes, which included length of stay (LOS), complications, and mortality. The required odds ratio (OR) was calculated, univariate and binary regression analyses were done, and a p-value of <0.05 was considered statistically significant.

Results

Of the 42 confirmed cases, 38.1% were males and 61.9% were females. The average age of male patients was 12.6 ± 11.2 years, while that of females was 22 ± 19.3 years. Of the patients, 64.3% were in the age group of 1-20 years. The clinical manifestations in descending order were fever (71.2%), skin rash (19.1%), cough (16.7%), vomiting (28.6%), altered sensorium (23.8%), abdominal pain (23.8%), loose stools (14.3%), seizures (14.3%), anasarca (9.7%), breathlessness (9.7%), and melena (7.1%). Eschar was noted in 38.1% of patients. Swelling of the body (6.7%) and lymphadenopathy (10%) were seen exclusively in children. The common laboratory abnormalities observed were leukocytosis in 34.3% of cases; thrombocytopenia in 68.8% of cases, of which 25% of patients had platelets < 50,000/mm^3^; and transaminitis in 87.5% of cases. The ratio of AST/ALT of more than one was seen in 89.3% of patients, while it was less than one in 10.7% of patients. The average C-reactive protein (CRP) level was 10.9 ± 6.3 mg/dL. The complications noted were acute respiratory distress syndrome (ARDS) (16.7%), meningoencephalitis (21.4%), septic shock (14.3%), capillary leak syndrome (26.2%), thrombocytopenia (68.8%), transaminitis (87.5%), myocarditis (4.8%), disseminated intravascular coagulation (2.4%), and hypocalcemia (11.9%). The average length of stay (LOS) was 8.1 ± 4.2 days. Twenty-four (57.2%) patients required transfer to the critical care unit (CCU) for managing various complications. There was no mortality in this series, giving rise to the case fatality ratio (CFR) of 0.

Conclusion

Scrub typhus is a reemerging cause of acute febrile illness. The highest number of cases were found during the post-monsoon period and in those with rural backgrounds. It presents with varying clinical manifestations with or without eschar. Hence, a high degree of suspicion along with a thorough clinical examination is needed to diagnose this condition. The disease responds dramatically to doxycycline. One must be aware of its complications and atypical presentations, as a timely diagnosis can reduce the morbidity and mortality associated with this disease.

## Introduction

Scrub typhus is caused by the mite-borne obligate intracellular bacterium *Orientia tsutsugamushi* belonging to the family Rickettsiaceae and transmitted by larval forms of *Leptotrombidium* mites called chiggers [1]. The disease, scrub typhus, occurs when infected mite larvae accidentally bite human beings. Over one million new infections are detected annually, and an estimated one billion people globally are at risk [2]. Without appropriate treatment, the case fatality rate (CFR) varies from 30% to 45% [3]. The disease burden in India is still unclear, but it is largely an overlooked problem. In India, after the early epidemic during the second world war in the states of Assam and Bengal, there has been a reemergence of cases in the recent years [4] from various states in India including Himachal Pradesh, Jammu and Kashmir, Uttaranchal, Rajasthan, Tamil Nadu, Kerala, Maharashtra, Bihar, Karnataka, West Bengal, and Meghalaya [5,6].

Early clinical manifestations of scrub typhus are nonspecific and may be confused for a viral illness, malaria, or bacterial illness. It often presents with fever, chills, headache, cough, nausea, vomiting, myalgia, and skin rash. However, at times, the disease may be complicated with myocarditis, pneumonia, acute kidney injury (AKI), meningoencephalitis, gastrointestinal (GI) bleeding, and multi-organ dysfunction syndrome (MODS), resulting in high mortality [6]. With early diagnosis and treatment, these complications and mortality rates can be considerably reduced.

A systemic review of the burden of scrub typhus cases in India, which included an analysis of published literature on scrub typhus in India over a 10-year period, was conducted by Devasagayam et al. [7]. The review included 138 hospital-based studies and two community studies. Of the 18,781 confirmed cases, 25.3% presented as acute undifferentiated febrile illness (AUFI). Among those with severe scrub typhus, 20.4% of patients required intensive care unit (ICU) admission, and 19.1% of patients eventually needed ventilation. MODS was reported in 33 published studies (17.4% of the total cases). The mortality among those with MODS was 38.9%, while the overall case fatality rate was 6.3%.

This review included 13 studies from Eastern India, of which seven were from West Bengal, four were from Odisha, and one each was from Bihar and Chattisgarh [7]. To the best of our knowledge, there were no reported studies from Jharkhand. In light of this, we undertook the present study at Tata Main Hospital (TMH) with the aim of studying the epidemiological features, clinical profile, laboratory features, and clinical outcomes in scrub typhus cases, identifying the predictors of disease severity, and studying their correlation with disease mortality.

## Materials and methods

This was a retrospective observational study. Patients in whom the diagnosis of scrub typhus was confirmed and who fulfilled the inclusion criteria from January 1, 2019, to December 31, 2021, were included in the study. The study population constituted of patients admitted to the medical wards and critical care unit (CCU) of Tata Main Hospital (TMH), Jamshedpur, which is a 1,020-bedded tertiary care hospital in Jharkhand. The study was given clearance by the Institutional Ethics Committee (IEC).

Inclusion criteria

The inclusion criteria included any febrile illness of ≥5 days with clinical features of myalgia, arthralgia, headache, and skin rash, with or without eschar at any site with a positive Weil-Felix agglutination test with antibody titers of 1:320 to Proteus OX-K antigen and/or an enzyme-linked immunosorbent assay (ELISA)-specific IgM titers against *O. tsutsugamushi* of ≥1:80 [5].

Exclusion criteria

Patients with other acute febrile illnesses such as malaria, dengue fever, chikungunya fever, enteric fever, and urinary tract infection, pregnant females, and cases where antibody tests were negative were excluded from this study.

Methodology

Demographic characteristics and clinical features such as a history of onset of illness, progression, duration of various symptoms, and vital parameters were noted from the medical records retrieved from the hospital management system (HMS). Blood investigations included complete blood picture including platelet count, liver function tests (serum bilirubin, alanine aminotransferase (ALT), aspartate aminotransferase (AST), alkaline phosphatase (ALP)), serum proteins (albumin and globulin), prothrombin time, international normalized ratio (INR), and renal function tests (blood urea and serum creatinine). These were done using automated biochemical analyzers on the day of admission and repeated every 72 hours until discharge. The findings of chest radiography, ultrasound examination of the chest and abdomen with pelvic and cardiac echocardiography, wherever available, were noted. The outcomes of the study included the length of stay (LOS), duration of intensive care unit (ICU) stay, duration of ventilator days, complications related to various organs, and mortality. The diagnosis was confirmed by performing a Weil-Felix reaction and/or IgM ELISA on serum samples using the scrub typhus detect test (InBios International, Seattle, USA), as per the manufacturer’s instructions.

Case definitions

Scrub Typhus Cases

These are patients with an acute febrile illness with or without an eschar confirmed by a serological test (specific IgM antibody/Weil-Felix reaction), as described above.

Case Fatality Rate (CFR)

The case fatality rate is the proportion of deaths among individuals diagnosed with scrub typhus.

Severe Scrub Typhus

Severe scrub typhus is defined by death or the presence of organ involvement as follows [7]: acute respiratory distress syndrome (ARDS), defined as the patient presenting with cough, dyspnea, oxygen saturation of <92% while breathing ambient air, and PaO2/FiO2 ratio < 300 with chest radiograph showing bilateral pulmonary infiltrates in the absence of cardiac dysfunction; significant transaminitis, defined as an elevation of aspartate aminotransferase (AST) and/or alanine aminotransferase (ALT) above two times the upper limit of normal (ULN) with or without serum bilirubin of 3 mg/dL and above (elevation of liver enzymes between two to five times the ULN, more than five to 10 times the ULN, and more than 10 times the ULN was considered mild, moderate, and severe transaminitis, respectively); acute kidney injury (AKI), defined as a serum creatinine level over 1.5 mg/dL with fall in urine output to <50 mL/hour; thrombocytopenia, defined as a platelet count of less than 100,000/mm^3^; myocarditis, defined as elevated creatine kinase (CKMB) and/or troponin T and abnormal electrocardiography; meningoencephalitis, defined as the presence of neurological symptoms, such as headache, altered sensorium, seizures, and neck stiffness with or without and CSF analysis, indicating elevated protein level in the absence of other obvious causes; and multi-organ dysfunction syndrome (MODS), defined as simultaneous or sequential dysfunction of two or more organ systems.

The confirmed scrub typhus patients were further divided into two groups [5]: group 1, those without severe scrub typhus, and group 2, those with severe scrub typhus.

Statistical analysis

Categorical variables were described in the form of numbers and percentages of patients, while continuous variables were described in the terms of means, medians, ranges, and standard deviations (SDs). The significance of the difference in means and odds ratios (ORs) were calculated using an independent sample t-test. Univariate analysis and binary logistic regression were done as required. The significance of the p-value was taken as p < 0.05.

## Results

**Figure 1:**
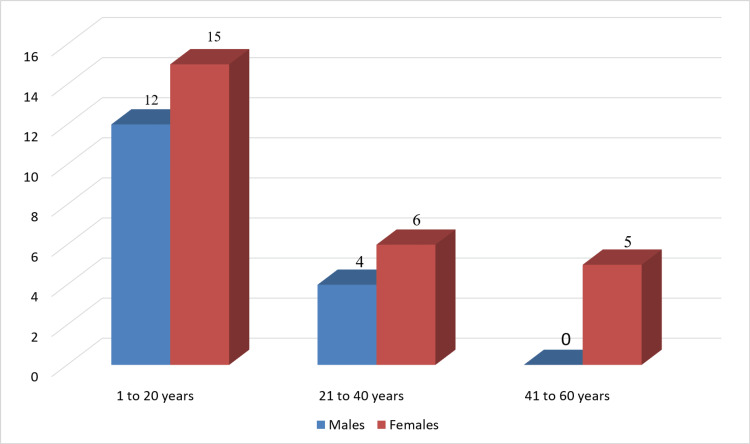
Age and sex distribution of the patients (N = 42)

Out of the suspected 65 cases, 42 cases were confirmed to be that of scrub typhus. Of the total of 42 patients, 16 (38.1%) patients were males and 26 (61.9%) patients were females, with the male/female ratio being 0.6:1. The average age of males was 12.6 ± 11.2 years, while that of females was 22 ± 19.3 years. Most of the patients (n = 27, 64.3%) were in the age group of 1-20 years and were females (n = 15, 55.6%). There were only 15 (35.7%) patients beyond the second decade of life (Figure 1). The oldest male patient was 36 years, while the oldest female patient was an elderly 65 years old.

Most of the cases (n = 27, 64.3%) were admitted in the post-monsoon period from October to December, while the rest were distributed sporadically throughout the year. Adult patients (n = 10, 83.3%) came from a rural background with a history of living close to bushes and wood piles, while 20 (66.7%) children had parents from rural unhygienic backgrounds.

**Figure 2:**
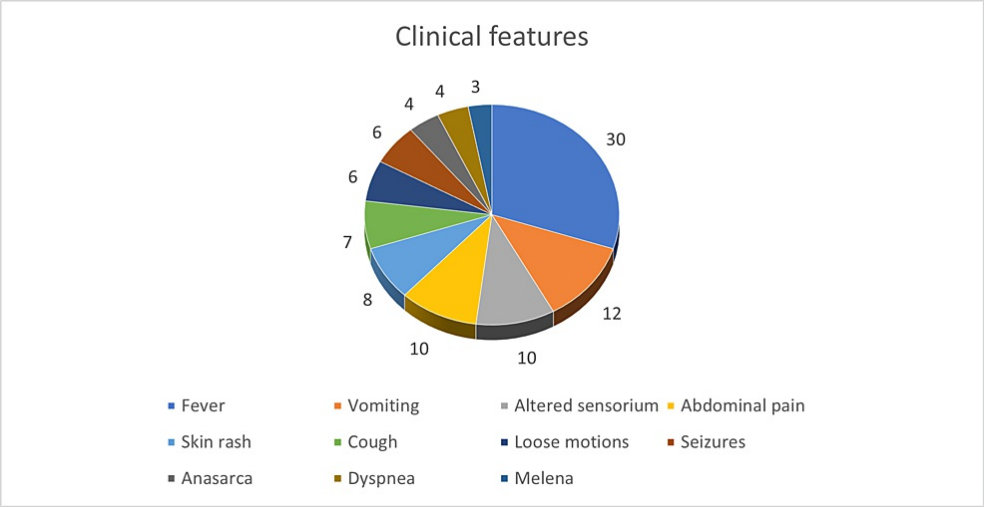
Clinical manifestations in the admitted patients (N = 42)

The average duration of fever was 9.5 ± 4.6 days. The most common symptom was fever, reported by 30 (71.4%) patients. It was associated with chills and rigors in 11 (36.7%) and headaches in eight (26.7%) patients. Fever was present for 5-10 days in 20 (66.7%) patients and more than 10 days in 10 (33.3%) patients. Skin rash was erythematous, maculopapular in nature, and seen in eight (19.1%) patients. In all patients, it appeared on the fourth to the sixth day of illness on the trunk and extremities and was associated with itching. The various clinical manifestations in the descending order were fever (n = 30, 71.2%), skin rash (n = 8, 19.1%), cough (n = 7, 16.7%), vomiting (n = 12, 28.6%), altered sensorium (n = 10, 23.8%), abdominal pain (n = 10, 23.8%), loose stools (n = 6, 14.3%), seizures (n = 6, 14.3%), anasarca (n = 4, 9.7%), breathlessness (n = 4, 9.7%), and melena (n = 1, 7.1%) (Figure 2).

**Figure 3:**
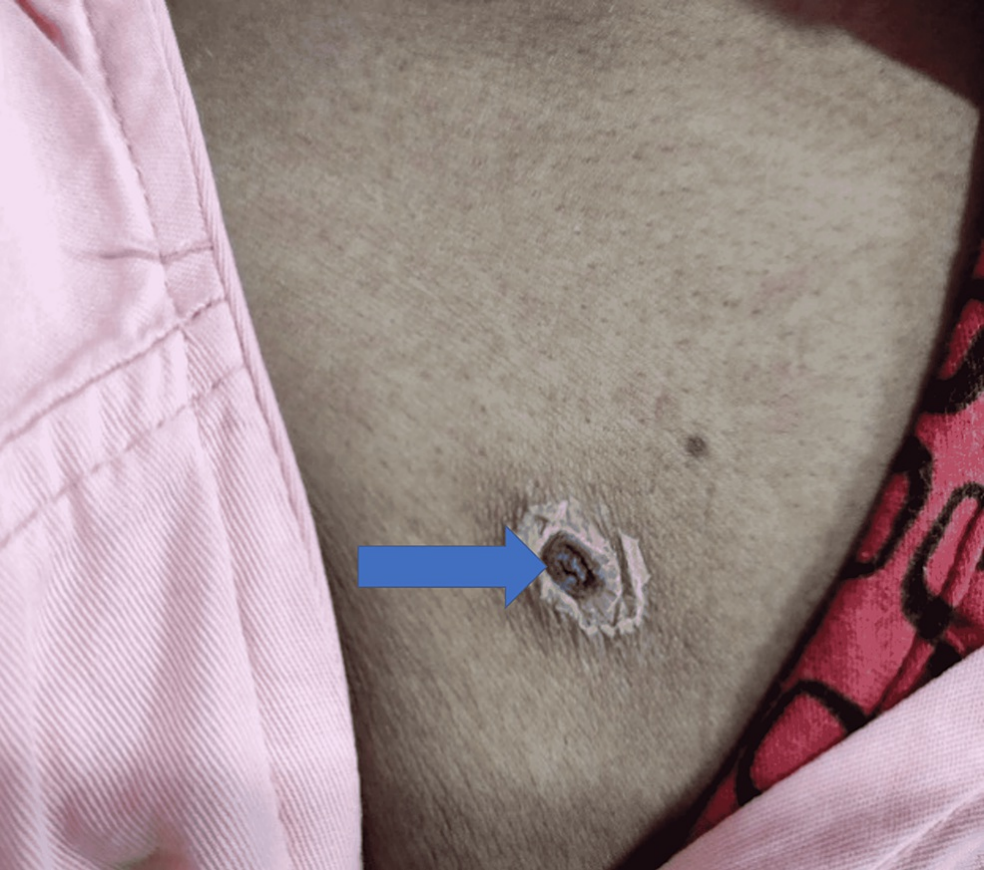
Eschar on the anterior abdominal wall

Eschar was noted in 16 (38.1%) patients. The various sites of eschar were the back in four (9.5%) patients, abdominal wall in four (9.5%), forearm in three (7.1%), thigh in two (4.8%), axilla in two (4.8%), and neck in two (4.8%) (Figure 3). Clinical findings on general examination included skin rash (n = 8, 19.1%), eschar (n = 16, 38.1%), lymphadenopathy (n = 5, 11.9%), hepatomegaly (n = 17, 40.8%), splenomegaly (n = 13, 30.9%), anasarca (n = 5, 11.9%), and hypotension (n = 2, 4.8%).

Three (7.2%) patients had comorbidities that included systemic lupus erythematosus (SLE), malaria, and tuberculosis. Dengue card test for NS1 antigen was falsely positive in seven (16.7%) patients, leading to an incorrect diagnosis of dengue fever initially. In all these cases, subsequently, dengue-specific IgM antibodies were not detected.

A comparison of the symptoms between children and adults is shown in Table 1. Children had a larger number of gastrointestinal (53.3% versus 41.7%) and central nervous system (CNS) (26.7% versus 16.7%) symptoms, respectively, when compared to adults. Swelling of the body (6.7%) and lymphadenopathy (10%) were seen exclusively in children. However, none of these observations were statistically significant (p < 0.05).

**Table 1:** Comparison of the symptoms between children and adults (N = 42)

S. no.	Symptoms	Children (<15 years) (n = 30)	Adults (≥15 years) (n = 12)	OR (95% CI)	p-value
1	Gastrointestinal (GI)	16 (53.3%)	5 (41.7%)	0.78 (0.23-2.6)	0.68
1a	Loose stools	2 (6.7%)	2 (16.7%)		
1b	Vomiting	6 (20%)	1 (8.3%)		
1c	Abdominal pain	8 (26.7%)	2 (16.7%)		
2	Respiratory	6 (20%)	3 (25%)	1.25 (0.26-5.8)	0.77
2a	Cough	5 (16.7%)	2 (16.7%)		
2b	Shortness of breath	1 (3.3%)	1 (8.3%)		
3	Central nervous system (CNS)	7 (26.7%)	2 (16.7%)	0.714	0.69
3a	Headache	4 (13.3%)	1 (8.3%)		
3b	Convulsion	3 (10%)	0 (0%)		
3c	Altered sensorium	1 (3.3%)	1 (8.3%)		
4	Skin rash	7 (23.3%)	1 (8.3%)	0.35 (0.39-3.22)	0.35
5	Swelling of the body	2 (6.7%)	0 (0%)	0.48 (0.021-10.9)	0.65
6	Lymphadenopathy	3 (10%)	0 (0%)	0.34 (0.16-7.25)	0.49

Laboratory investigations

Complete data were available only for 32 (76.2%) patients. While the white blood cell (WBC) count was normal in 16 (50%) patients, leukocytosis was observed in 11 (34.3%) cases with neutrophilic leukocytosis seen in nine (84%) and leucopenia in five (15.6%) patients considering normal range as 4,550/mm^3^ to 11,000/mm^3^. The lowest leucocyte count seen was 2,000/mm^3^, while the highest count was 39,100/mm^3^. The average leucocyte count was 10,234.3 ± 6,864.1/mm^3^. Lymphocytosis was noted in eight (25%) patients and monocytosis in 10 (31.3%) patients. The average hemoglobin level was 9.7 ± 1.8 g/dL, while the average platelet count was 115,375 ± 65,069/mm^3^. Thrombocytopenia was seen in 22 (52.4%) patients. The severity of thrombocytopenia was as described in the complications.

Transaminitis was observed in 28 (87.5%) of the cases, of which mild transaminitis was seen in 20 (71.8%) patients, moderate elevation was seen in six (21.4%) patients, and severe transaminitis was noted in two (6.3%) patients. The average values of ALT and AST were 145.8 ± 244.01 U/L and 179.8 ± 142.3 U/L, respectively, while that of serum bilirubin was 0.9 ± 0.8 mg/dL. The ratio of AST/ALT of more than one was seen in 25 (89.3%) patients, while it was less than one in three (10.7%) patients. Two (4.8%) patients had mild clinical jaundice. The average C-reactive protein (CRP) level was 10.9 ± 6.3 mg/dL, while the highest level noted was 23.2 mg/dL.

Complications

Thrombocytopenia

Twenty-two (68.8%) patients had thrombocytopenia, of which eight (25%) patients had platelets < 50,000/mm^3^ and one (3.1%) patient had severe thrombocytopenia. The lowest platelet count noted in the study was 9,000/mm^3^, with patients having ecchymotic patches over the forearm, petechial hemorrhages, and subconjunctival and gum bleeding. One (3.1%) patient with a platelet count of 38,000/mm^3^ presented with melena. The rest of the patients with thrombocytopenia did not have any bleeding manifestations.

Meningoencephalitis

Nine (28.1%) patients had features of meningoencephalitis and presented with irritability and altered sensorium. Of these, six (14.3%) patients had seizures, focal in five (11.9%) patients and generalized in one (2.4%) patient. One (2.4%) child developed status epilepticus. Neck stiffness was found in one (2.4%) patient. Cerebrospinal fluid (CSF) examination done in all nine patients showed an average of 87 cells, mostly lymphocytes, with proteins of 122.1 ± 44.5 mg/dL, sugar of 58.1 ± 10.1 mg/dL, and adenosine deaminase (ADA) level of 4.2 ± 1.1 U/L. The maximum cells seen were 200 lymphocytes, and the maximum CSF protein level was 213.4 mg/dL.

Septic Shock

Six (14.3%) patients were in septic shock and required inotropes, of which four (9.5%) needed double (adrenaline and noradrenaline) inotropes, while two (4.8%) were on single (noradrenaline) inotrope. Three (7.1%) patients had hypotension and needed an intravenous bolus of crystalloids for correction.

Polyserositis (Capillary Leak Syndrome)

Evidence of bilateral pleural effusion with ascites was seen in 11 (26.2%) patients. The effusion was mild to moderate and had resolved in six (14.3%) patients at the time of discharge. One (2.4%) patient also had mild pericardial effusion. All these patients had low serum albumin levels (mean albumin level: 2.3 gm/dL).

Myocarditis

Echocardiography of two (4.8%) patients revealed global left ventricular (LV) hypokinesia suggestive of myocarditis. While one patient had an LV ejection fraction (LVEF) of 50%, the other had moderate LV dysfunction (LVEF: 40%). Both were seen in the pediatric group.

Acute Respiratory Distress Syndrome (ARDS)

**Figure 4:**
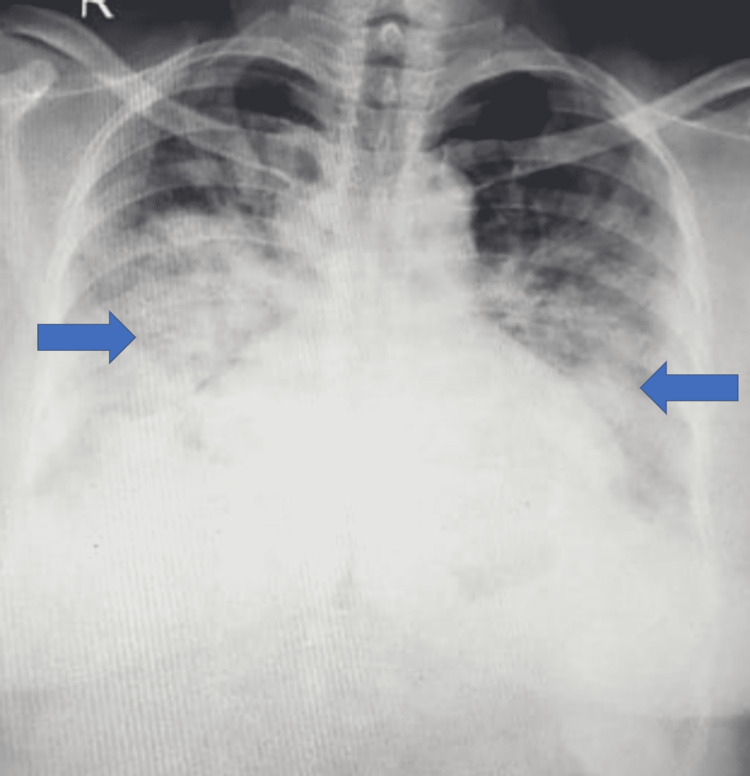
Chest radiograph showing bilateral mid and lower zone opacities (arrows)

Seven (16.7%) patients developed ARDS, of which four (9.5%) developed severe ARDS, while three (7.1%) had mild ARDS. Two (4.8%) required mechanical ventilation, and one (2.4%) each needed high-flow nasal oxygen (HFNO) at 12 L/minute and noninvasive ventilation (NIV). Chest radiographs in these patients revealed bilateral opacities in the mid and lower zones (Figure 4).

Transaminitis

Most (n = 20, 71.8%) had mild transaminitis, while clinical jaundice was evident only in two (4.8%) patients. The maximum levels of ALT, AST, and serum bilirubin observed were 698 U/L, 1,308 U/L, and 3.7 mg/dL, respectively. None of the patients developed acute liver failure (ALF).

Hypocalcemia

It was found in five (11.9%) cases, all in the pediatric age. One patient had low levels of serum vitamin D. It was not known whether this was the direct complication of the disease or preexisting before hospital admission. All were given calcium supplements.

Disseminated Intravascular Coagulation (DIC)

Only one (2.4%) patient had DIC. He had a platelet count of 9,000/mm^3^, low serum fibrinogen of 120 mg/dL, high INR of 1.9, and fibrin degradation products (FDP) of 3,200 mg/dL.

MODS

It was found in seven (16.7%) patients in our study. Of these, six (14.3%) patients had only two-organ failures (2OF) and only one (2.4%) had three-organ failure. Of the six patients with 2OF, three (7.2%) patients had combined dysfunction of cardiovascular (CVS) and respiratory systems, two (4.8%) had dysfunction of respiratory and CVS systems, and one (2.4%) each had dysfunctions of hepatic with hematological systems. While respiratory dysfunction was found in seven (16.7%) patients, CVS dysfunction was noted in six (14.3%) and CNS dysfunction in two (4.8%) patients. None of the patients had renal involvement. Hepatic involvement was seen in 28 (89.3%) cases, but dysfunction was observed only in two (7.1%) cases.

**Figure 5:**
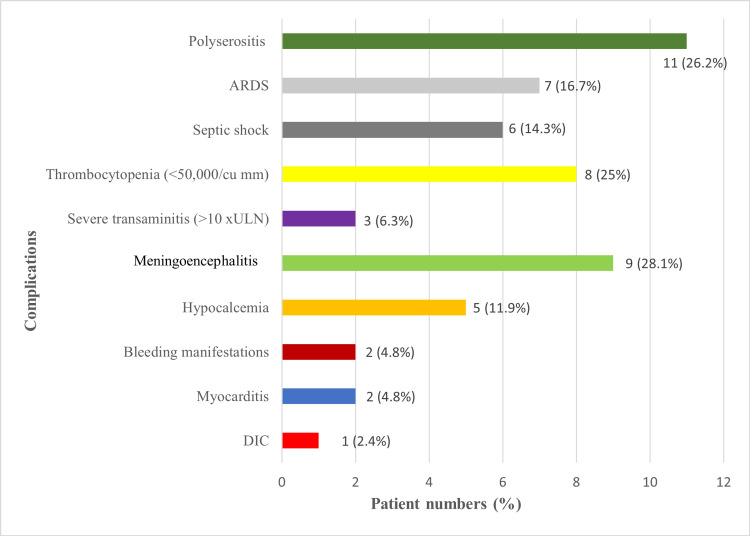
Complications observed in the study (N = 42) DIC: disseminated intravascular coagulation, ULN: upper limit of normal, ARDS: acute respiratory distress syndrome

The abovementioned complications are depicted in Figure 5.

Outcomes

The average length of stay (LOS) was 8.1 ± 4.2 days. Twenty-four (57.2%) patients required transfer to the critical care unit (CCU) for managing various complications. There was no mortality in this series, giving rise to the case fatality ratio (CFR) of 0. The complications noted were described above. As there was no mortality in our study, we tried to find only the predictors of disease severity (Table 2).

**Table 2:** Predictors of the disease severity *p-value calculated from mean and standard deviation (SD)

S. no.	Parameters	Mild disease (mean ± SD) (n = 14)	Severe disease (mean ± SD) (n = 28)	p-value*
1	Age (years)	20.7 ± 19.03	15.5 ± 15.7	0.3516
2	Duration of fever (days)	7.4 ± 3.3	8.1 ± 3.5	0.5372
3	Total leucocyte count (/mm^3^)	7,341.2 ± 2,170	13,792 ± 8,704.5	0.0097
4	Platelet count (/mm^3^)	155,312 ± 54,345.8	71,617 ± 46,716.1	0.0001
5	Alanine transaminase (U/L)	54.9 ± 23.8	267.3 ± 319.6	0.01
6	Aspartate transaminase (U/L)	96.1 ± 51.5	292.6 ± 136.4	0.001
7	C-reactive protein (CRP)	4.5 ± 1.7	13.2 ± 5.5	0.0001
8	Serum albumin (mg/dL)	3.4 ± 1.2	2.3 ± 1.1	0.06

Among the biochemical parameters, serum albumin, CRP levels, and ALT and AST levels on admission along with total leucocyte count and platelet count were significantly associated with severe disease and MODS (Table 2). Age and the duration of fever were not related to disease severity. On applying binary logistic regression, the same parameters were found to be statistically significantly associated with disease severity (Table 3).

**Table 3:** Logistic regression analysis for disease severity and MODS MODS: multi-organ dysfunction syndrome, OR: odds ratio, CI: confidence interval

S. no.	Study parameters	OR	95% CI	p-value
1	Total leucocyte count (/mm^3^)	1.0004	1.0001-1.0007	0.0147
2	Platelet count (/mm^3^)	1.0	1-1	0.0041
3	Alanine aminotransferase (U/L)	1.054	1.0167-1.0929	0.0043
4	Aspartate aminotransferase (U/L)	1.033	1.0075-1.0596	0.011
5	Serum C- reactive protein (mg/dL)	2.0085	1.1700-3.4301	0.0113
6	Serum albumin (g/dL)	2.0034	1.1230-3.5420	0.0110

## Discussion

Scrub typhus is a vector-borne zoonotic infection and is one of the commonest rickettsial infections worldwide. The disease is endemic in South Asia, Southeast Asia, East Asia, the Pacific Islands, and Northern Australia (“tsutsugamushi triangle”) [1]. However, cases also have been reported from Chile, Peru, Africa, and the Arabian Peninsula. In India, there has been a reemergence of cases in recent years from various states [4]. The analysis of strain DNA by immunological methods suggests that the Karp strain causes about 50% of all cases in the endemic regions, including South Asia [8].

A study of seasonal distribution showed higher incidence in the months from October to December. Similar observations were made by Islam et al. [8] and Ngulani et al. [9] in their studies from Eastern India. However, the highest number of cases were reported from July to October (during monsoon) in the study by Gurung et al. [10] from Sikkim and in yet another study by Sharma et al. from Darjeeling [11].

Varghese et al. reported that the mean age of scrub typhus patients in the southern part of India was 36.5 years (range: 12-75 years) [4], while the study by Sinha et al. included patients from three to 78 years [12]. In our study, most of the patients (64.3%) were in the age group of 1-20 years and were females. As in our study, the study by Islam et al. revealed that 61.2% of the cases were in the age group of 1-14 years. Of the cases in their study, 62.3% were males [8]. Male predominance was also seen in the study by Sharma et al. [11].

The disease often manifests as an acute undifferentiated febrile illness in 35%-50% of cases [2,4]. It starts with abrupt onset of fever associated with a sore throat, cough, myalgia, headache, skin rash, and an eschar at the site of inoculation after a variable incubation period of 5-14 days after the person is bitten by infected chiggers. In a systematic review of the burden of scrub typhus in India by Devasagayam et al., some of the common presenting symptoms of confirmed cases in the descending order were fever (34.7%), eschar (22.1%), headache (18.2%), nausea/vomiting (17.1%), abdominal pain (10.5%), breathlessness (10.4%), cough (10.4%), jaundice (5.6%), and seizure (2.7%) [7]. The study by Gurung et al. found pedal edema (55.6%), facial puffiness (51.8%), and rash (44.4%) predominantly in children [10]. Similar symptoms were noted in our study.

Eschar was found in 46% of patients by Vivekanandan et al. in Pondicherry [3]. Although it may be found in other conditions, its presence in the setting of unexplained febrile illness is pathognomonic of scrub typhus. It begins as a painless papule that evolves into a dark-colored scab-like scar known as eschar. It often goes unnoticed as it is painless and does not itch. It is seen with variable frequency from 9.5% in North India [1] to 90% in a Korean study [5]. In our study, it was found in 16 (38.1%) patients and in 45.5% of cases in the study by Chrispal et al. from South India [2]. However, it was not found in a single case in a study from Northwestern India by Sinha et al. [12]. Such a wide variation probably reflects the inadequate search for the eschar or different feeding habits of various strains of the organism.

Laboratory findings highly suspicious of scrub typhus include elevated transaminases, thrombocytopenia, and leukocytosis. Chogle et al. [13] reported that the positive predictive value for scrub typhus increases to 80% if this triad of findings is present [14]. Leukocytosis was found in 46% of cases in the study by Varghese et al. [6], 34.3% of the patients in our study, only 10.5% of patients in the study by Liu et al. [15] in northern China, and 16.6% of cases in the study by Sinha et al. [12] in Northwestern India. Data published from northern China revealed thrombocytopenia ranging from 4.6% to 48.9% [15]. It was reported in 79% of cases in the study by Varghese et al., of which only 19.5% had severe thrombocytopenia (<30,000/mm^3^), while the rest had mild thrombocytopenia [6]. It was identified in 52.9% of cases in our study, while its incidence was as low as 10% in a study from Puducherry by Vivekanandan et al. [3]. It was reported in 85.7% of cases in the study by Sinha et al., but none had platelets below 50,000/mm^3^ [12]. It was the most common hematological abnormality noted by Ngulani et al. in 30.5% of cases from Manipur [9].

The severity of the disease depends on the virulence of the strain, susceptibility of the host, or both. Severe scrub typhus is associated with various organ involvement.

Pulmonary manifestations

These include bronchitis and interstitial pneumonia with or without pulmonary vasculitis, which may progress to ARDS. Wang et al. reported that about 11% of scrub typhus patients progressed to ARDS with a mortality rate of 25% [16]. In the study by Tsay et al., older age, thrombocytopenia, the occurrence of early pneumonia, and delay in diagnosis were identified as risk factors for ARDS [17]. In their four-year experience, 36% (12/33) of scrub typhus patients developed pneumonia, of which 42% progressed to ARDS and two (16%) expired. It was reported in 20.5% of cases by Devasagayam et al., of which 19.1% required mechanical ventilation [7]. In a five-year study by Varghese et al., which included 623 patients, respiratory system dysfunction occurred as a part of MODS in 76.9% of cases. ARDS was seen in 33.2% of cases, while ventilator support was required in 68.9%. The case fatality rate in those with ARDS was 18% [6]. In a recent review on scrub typhus, John and Varghese reported that pneumonia progressing to ARDS was seen in more than one-third of patients requiring hospitalization and was one of the common complications [18].

Various studies have reported abnormal chest radiographs in 59%-72% of patients [19,20]. Abnormal chest X-rays were found in nine (21.4%) patients in our study. Seven (16.7%) patients with ARDS had bilateral mid and lower zone opacities, and two (4.8%) had left lower zone opacities. The typical radiological findings described are diffuse bilateral reticulo-nodular opacities (up to 40%), septal lines, and hilar adenopathy, while ground-glass opacities, airspace consolidation, and pleural effusions are relatively uncommon [2,21].

Cardiac manifestations

These include myocarditis, heart failure, pericarditis, and electrocardiogram (ECG) abnormalities [18]. Myocarditis was documented in only two (4.8%) patients in this study, while it was reported in 1.1% of the cases by Chrispal et al. [2]. One study by Karthik et al. involving 80 patients found reduced left ventricular ejection fraction (LVEF) in 30% of patients and elevated troponin T in 61.7% of cases [22]. Although sinus tachycardia is the most common ECG abnormality described in the literature [18], Ngulani et al. described bradycardia in 6/176 patients (3.4%) with scrub typhus from Manipur [9].

Renal manifestations

The incidence of acute kidney injury (AKI) varies from 18% [4] to 66.4% [1]. It was reported in 19.6% and 26.1% of cases by Chrispal et al. [2] and Sinha et al. [12], respectively. None of the patients in our series had renal complications. It is considered to be relatively uncommon in mild cases and is seen in severe forms associated with severe forms associated with sepsis or MODS, with 3 to 10% of cases requiring hemodialysis. Renal failure may occur due to systemic vasculitis, rhabdomyolysis, hypoperfusion of the kidneys secondary to shock, microangiopathy, and possible direct invasion of the renal tubular cells resulting in acute tubular necrosis [23].

Gastrointestinal and hepatobiliary manifestations

Gastrointestinal symptoms (nausea, vomiting, and abdominal pain) were reported in 54% and 37% of cases, respectively, by Varghese et al. [4] and Chrispal et al. [2]. Various gastrointestinal (GI) manifestations include GI vasculitis, acute acalculous cholecystitis, acute pancreatitis, pancreatic pseudocyst formation, hepatocellular cholestasis, pericholangitis, and ALF [8,24]. Hepatic involvement is probably related to the predilection of the organism for liver sinusoidal endothelial cells [24]. Hepatomegaly was reported in 37%-59.5% of cases in various studies [24,25], while it was found in 40.8% of the patients in our study. Mild hepatitis was reported in 87% and 95.2% of the patients by Varghese et al. [6] and Chrispal et al. [2], respectively. Our study also reported a high degree of involvement of the liver (71.8%), although mild. Chrispal et al. found significant elevations of liver enzymes (>1,000U/L) in those with septic shock and MODS [2]. Only one (2.4%) patient in our study had an ALT elevation of more than 1,000U/L. Two cases of ALF due to scrub typhus have been reported by Deepak et al. [25]. However, there were none with ALF in our study. The study by Ngulani et al. observed that the elevation of ALT was more than that of AST [9], similar to that reported by Yang et al. [26]. However, we observed that the ratio of AST/ALT was more than one in 25 (89.3%) patients, while it was less than one in only three (10.7%) patients. This was similar to that reported by Sivarajan et al. from Manipur [27].

Central nervous system (CNS) manifestations

Several CNS manifestations described include aseptic meningitis, meningoencephalitis, Guillain-Barré syndrome, acute disseminated encephalomyelitis, movement disorders, cerebellitis, cortical vein thrombosis, opsoclonus and myoclonus, transverse myelitis, bilateral optic neuritis, and various neuropsychiatric manifestations that are related to CNS vasculitis resulting in ischemia and micro-infarctions [28]. Clinical symptoms vary from agitation to coma. Aseptic meningitis and meningoencephalitis are relatively common and were seen in 20.8% of patients in the study by Chrispal et al. [2], 23.3% of cases in the study by Varghese et al. [6], and 21.8% in our study, while a lower incidence of 9.5% was reported by Mahajan et al. [1]. CSF findings may resemble that of tubercular or viral meningitis as described in the study by Sivarajan et al. [27].

Septic shock

It was seen in 16.7% of our cases, 4.7% in the study by Sinha et al. [12], 13.8% in the study by Chrispal et al. [2], and 23.1% in the study by Varghese et al. [6]. It was associated with higher mortality in all studies other than ours.

MODS

Of the cases, 16.7% had MODS in the study by Sinha et al. [12], while it was present in 34% of patients in the study by Varghese et al. [6] and 42.8% in the study by Kim et al. [5]. It was observed in seven (16.7%) patients in our study. Of these, six (14.3%) patients had only two-organ failures and only one (2.4%) had three-organ failure. In the study by Takhar et al., 48.5% had three organ systems involved, while 30% of patients had evidence of dysfunction of five organs during the hospital stay [29].

Predictors of disease severity

In our study, age and the duration of fever did not correlate with disease severity. High transaminases, low platelet count, leukocytosis, low serum albumin, and high CRP were found to be predictors of severe disease. The study by Sivarajan et al. from Manipur revealed that platelets < 100,000/mm^3^, serum creatinine > 1.5 mg/dL, transaminase (AST, ALT, or both) > 500 U/L, and serum bilirubin > 3 mg/dL were associated with MODS (p < 0.001) and mortality (p < 0.05) [27].

As the serological tests for scrub typhus become positive during the second week of the illness, they cannot provide early diagnosis, and specific treatment may be delayed while awaiting reports, resulting in a high complication rate and mortality. The case fatality rate in various studies was as low as 2%-16.7% [12]. However, with increasing awareness and early treatment, there is now a trend toward decreasing mortality in published studies from India. A recent study from Manipur of 176 confirmed cases of scrub typhus by Ngulani et al. [9] revealed a mortality rate of 4.5%. There was no mortality in our study. This could possibly be due to the low virulence of the infecting organism, the younger population involved, and timely treatment.

The drug of choice in the treatment of scrub typhus is doxycycline with therapeutic response to doxycycline being used as a diagnostic test. The recommended dose is 100 mg twice daily orally in adults and 2.2 mg/kg body weight twice daily in children weighing less than 40 kg and 100 mg twice daily in those weighing above 40 kg [30]. The optimum duration of therapy is seven days and 14 days in case of complicated cases with MODS. Other antibiotics that may be used in doxycycline-resistant cases include macrolides such as azithromycin and rifampicin. Azithromycin is preferred in women during pregnancy, in whom doxycycline is contraindicated [31]. All our patients responded to doxycycline with resolution of symptoms within seven days of starting azithromycin.

This study highlights the need for increased awareness of rickettsial infections in Eastern India. We suggest that the diagnosis of scrub typhus should be based on a high index of suspicion, thorough clinical evaluation, and a constellation of laboratory parameters, as mentioned above. Early empirical use of doxycycline should be considered in the absence of contraindication to reduce the high mortality observed with the disease.

## Conclusions

Scrub typhus is prevalent in this part of the country and should be considered in the differential diagnosis of patients presenting with acute febrile illnesses with leukocytosis, thrombocytopenia, renal impairment, abnormalities in liver function tests, pneumonia, or ARDS. A history of rural background with agriculture-related activity should raise the suspicion. A careful search for an eschar, particularly in the hidden areas, is essential as it points toward the diagnosis. However, the eschar may not be present in a large number of cases, and its absence should not make the clinician exclude the diagnosis of scrub typhus. Developing awareness of this disease, especially in endemic areas, is essential among clinicians for the early detection of the disease to prevent complications. Empirical therapy with doxycycline should be started when the index of suspicion is high as it may be lifesaving.
